# Fabrication and performance enhancement of an In_2_O_3_/BiVO_4_ heterojunction for *N*-butanol gas sensing applications

**DOI:** 10.1039/d4ra04949g

**Published:** 2024-12-17

**Authors:** Xiang-Bing Li, Shuang Sun, Xiang Hu, Qian-Qian Zhang, Cheng Gao, Hui Zhou, Bao-Xu Wu, An-Qi Wang, Wen-Yao Hu, Yi-Jia Wang, Li-Xiong Yang, Bin Yang, Wen-Ke Li, Hong-Hong Xu

**Affiliations:** a Engineering Research Center of Integrated Circuit Packaging and Testing, Ministry of Education, Department of Physics, Tianshui Normal University Tianshui Gansu 741001 China lixiangbing@tsnu.edu.cn

## Abstract

Butanol, a highly toxic volatile organic compound, poses significant health risks. Consequently, the creation of efficient gas-sensitive materials for butanol detection holds substantial practical significance. This study employed a secondary hydrothermal technique to synthesize In_2_O_3_, BiVO_4_, and their composite In_2_O_3_/BiVO_4_. Notably, the In_2_O_3_/BiVO_4_ composite exhibited a threefold enhanced response, short desorption time and low operating temperature compared to pure BiVO_4_. Moreover, the composite demonstrated improved selectivity, certain moisture-proof performance, and prolonged stability. The synthesis strategy, which entailed growing microspherical In_2_O_3_ on BiVO_4_, led to structural modifications, enhanced surface area, increased oxygen adsorption capacity, an enlarged optical bandgap, and improved anti-interference ability of the device. As a result, the formation of an n–n heterojunction between In_2_O_3_ and BiVO_4_ in the composite material translates into an outstanding butanol sensing device.

## Abstract 2

In_2_O_3_/BiVO_4_ preparation procedure.

## Graphical abstract

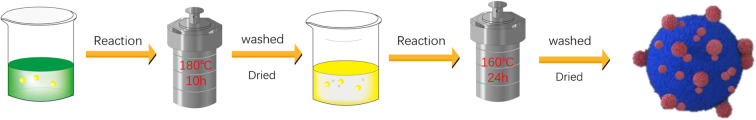

## 1. Introduction

With the gradual development of science and technology, the demand for various materials in chemical industry, materials science, biology, medicine and other fields is increasing day by day. Moreover, the production process of materials is accompanied with the release of toxic and harmful gases. Butanol is a colorless transparent liquid with a unique odor and is slightly soluble in water. It is widely used as a solvent, extraction agent and surfactant and plays an important role in the chemical industry.^1^ However, owing to its irritation and anesthetic properties, it may cause dizziness and drowsiness as well as damage the eyes, skin and even the respiratory system.^2^ Because of its wide range of uses and great potential to harm the human body, it is of great significance to develop a gas sensor that can effectively detect butanol.

Metal oxide semiconductor (MOS) sensors, recognized for their economic viability and manufacturing simplicity, have piqued significant interest owing to their practical applications.^3^ The judicious selection of gas-sensitive materials is at the heart of improving sensor performance, which is a very important area of research.^4^ Some of the good gas-sensitive materials include SnO_2_, ZnO, ZnFe_2_O_4_, and GaN.^5–7^ Although MOS sensors are highly reactive to gases, challenges remain, such as high operating temperatures and insignificant selectivity. Among the vanadate derivatives, luminescent rare earth vanadate (REVO_4_) stands out, and its contribution to photocatalytic devices is multifaceted, especially in the field of gas sensing applications, which has great application prospects. Bismuth vanadate (BiVO_4_) is commonly utilized in photocatalysis,^8^ harnessing solar energy, wastewater treatment, water purification, and hydrogen production; it also has high thermal and chemical stability and good gas sensitivity.^9,10^

Mozharov *et al.* discussed the gas sensing behavior, active sites, and sensing mechanisms of different polymorphic forms of BiVO_4_.^11^ In a study by Pei *et al.*, a glycol-sensitive gas sensor was obtained by adjusting the pH of BiVO_4_ with a response of 90.^12^ Chen *et al.* improved the response by decorating Pd nanoparticles onto the (010) face of the BiVO_4_ decahedron.^13^ Wang *et al.* prepared BiVO_4_ microspheres using a simple hydrothermal method and adjusted the molecular weight of PEG to change the morphology of the microspheres; they found that the higher the molecular weight, the more favorable was the formation of porous spherical nanostructures. Further, maximum yield was attained at 340 °C.^14^ Luo *et al.* prepared BiVO_4_ nanoplates by adding an active agent. A high response butanol gas sensor was obtained at 260 °C.^10^

Efforts to enhance sensor performance commonly involve strategies such as heterostructure synthesis, shape manipulation, incorporation of dopants, and augmentation with noble metals. However, the full potential of BiVO_4_ as a gas sensor is yet to be fully realized.^8^ To enhance the electronic conductivity of BiVO_4_ and expedite its response times, we strategically employed In_2_O_3_ as a composite booster.^15,16^ Renowned for its unparalleled conductivity compared to SnO_2_, ZnO, and Fe_2_O_3_, In_2_O_3_ also boasts remarkable optical and electrical attributes.^17–19^ It exhibits significant promise in various applications, including gas sensing, display technologies, photocatalysis, light-emitting diodes, and photoelectric detection.

In this research, we synthesized In_2_O_3_/BiVO_4_ nanocomposites using a synergistic hydrothermal method, significantly boosting the gas sensor's sensitivity and shortening its recovery time. Extensive characterization techniques, such as XRD and SEM, were applied. Comparative tests revealed the extraordinary selectivity (100 ppm butanol), high response rate (*S* = 45%), lower operating temperatures (210 °C), and enhanced adsorption–desorption efficiency. A comprehensive investigation focused on enhanced sensing attributes linked to oxygen vacancies, surface area, electron–hole recombination, and optical bandgap.

## 2. Experimental

### 2.1 Preparation of BiVO_4_

To prepare a BiVO_4_ mixture, 43 mL of deionized water was first mixed with 17 mL of concentrated HNO_3_ (18.56 mol L^−1^) to obtain 60 mL of diluted HNO_3_ solvent at a concentration of 5.2 mol L^−1^. Next, 5 mmol Bi(NO_3_)_3_·5H_2_O and 5 mmol NH_4_VO_3_ were sequentially introduced into this solvent while stirring vigorously until a homogeneous yellow suspension was formed. Then, ammonia was carefully added dropwise through a dropper to regulate the pH level. Subsequently, the adjusted solution was transferred into a 100 mL Teflon-coated stainless steel autoclave, maintaining a temperature of 180 °C for a period of 10 hours. Following the reaction, the contents of the autoclave were centrifuged, and then, the yellow precipitate thus obtained was washed with ethanol and deionized water until a clear supernatant was obtained. The residue was dried at 60 °C for 4 hours, followed by annealing it in a furnace at 500 °C for 3 hours to achieve the final product.

### 2.2 Preparation of the In_2_O_3_/BiVO_4_ composite sample

InCl_3_·4H_2_O (2 mmol), citric acid (5 mmol), and urea (30 mmol) were mixed in a water–glycol blend (30 mL water and 30 mL EG). Next, a 0.5 g BiVO_4_ dispersion was homogenized through magnetic stirring for 50 minutes. The mixture was transferred to a 100 mL Teflon-coated stainless steel autoclave and then heat treated at 160 °C for 24 hours. After cooling to room temperature naturally, the product was collected *via* centrifugation at 9000 rpm, thoroughly washed with distilled water and ethanol, and finally dried at 60 °C for 12 hours. The obtained composite sample was annealed at 400 °C, affording In_2_O_3_, BiVO_4_ and the final composite.

### 2.3 In_2_O_3_/BiVO_4_ sensor preparation and gas sensing detection

**Fig. 1:**
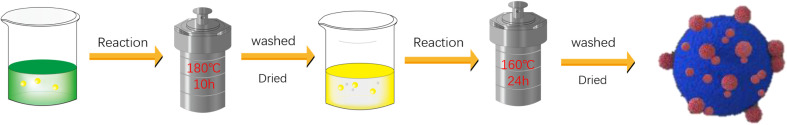
In_2_O_3_/BiVO_4_ preparation procedure.

First, a disposable glass substrate was employed. A powdered sample was homogenously mixed with water in a 4 : 1 weight ratio, forming a smooth paste. This paste was then uniformly applied onto a 0.5 mm thick and 4 mm long ceramic tube, which featured gold electrodes and four platinum leads. A nickel–chromium heating wire was inserted inside the tube, allowing for temperature control by varying heating voltage (*V*_H_). Subsequently, the sensor coated with the In_2_O_3_/BiVO_4_ composite underwent an aging process. It was aged for 48 hours at 120 °C to ensure optimal stability, as depicted in Fig. 1.

**Fig. 2:**
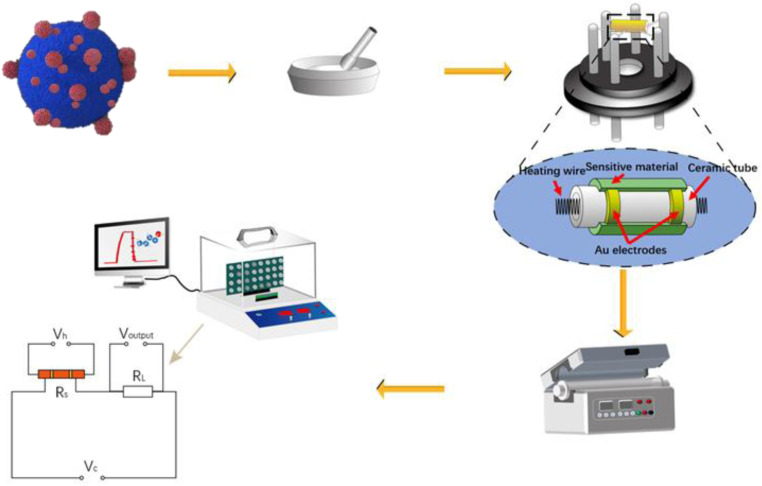
Sensor preparation process and test process.

In the preliminary stage of gas-sensitivity evaluation, the testing setup is initially ventilated using a fume hood with pristine air. Subsequently, an exact volume of test liquid is precisely administered to the vaporization platform *via* a micropipette. Rapid evaporation ensues, swiftly filling the chamber and triggering a response that is captured by the sensor's signal detection device. As depicted in Fig. 2, this process induces a change in the sensor's resistance, which is in adherence to the conversion formula for calculating gas concentration from the liquid phase:1
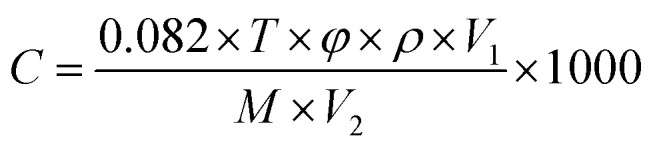
where *C* (in parts per million) is the desired gas concentration, with a fixed gas volume fraction of 0.082. The equation also depends on the liquid's density, where *V*_1_ represents liquid volume and *V*_2_ denotes gas chamber volume. Additionally, the molecular weight of the liquid plays a role. Gas response (*S*) is mathematically expressed as the ratio of resistance in the air (*R*_a_) to that in test gas (*R*_g_):

2 *S* = *R*_a_/*R*_g_

Sensor resistance is calculated according to the following formula:

3 *R*_s_ = (*V*_C_ − *V*_output_)*R*_L_/*V*_output_,

where *V*_c_ is bias voltage (5 V), *R*_L_ is load resistance, and *V*_op_ is output voltage.

### 2.4 Characterization methods

XRD and XPS analyses were performed on a Rigaku Ultima IV diffractometer and Thermal Science K-Alpha photoelectron spectrometer to determine the structural and elemental makeup of the composite. SEM and TEM inspections were carried out through a Zeiss Sigma 300 scanning electron microscope and JEOL JEM-F200 transmission electron microscope for morphological investigation. The surface area and pore size were assessed with a 3H-2000PS4 analyzer. Optical properties were studied *via* UV-visible spectroscopy and fluorescence spectroscopy using a U-3900H spectrophotometer and F-7100 fluorescence spectrophotometer, respectively. Lastly, the gas sensing capabilities of In_2_O_3_/BiVO_4_ composite materials were rigorously tested using a WS-30B gas sensor tester.

## 3. Results and discussion

### 3.1 Crystal structure analysis of samples

**Fig. 3:**
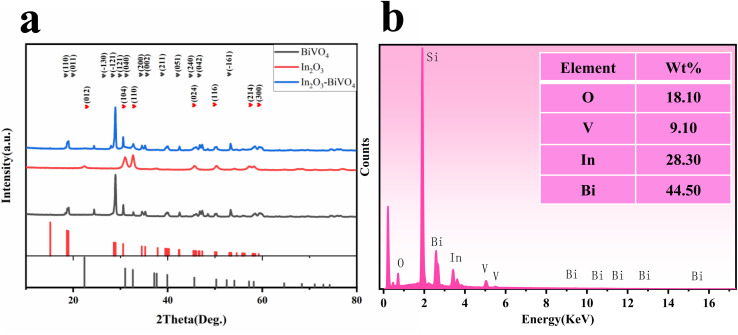
(a) XRD pattern of In_2_O_3_/BiVO_4_; (b) EDS profile of In_2_O_3_/BiVO_4_.

The crystal phase of the sample was determined *via* XRD diffraction and energy spectrum analysis. Fig. 3(a) shows the XRD spectra of In_2_O_3_, BiVO_4_ and In_2_O_3_/BiVO_4_ composite samples. In Fig. 3(a), the grey line represents the diffraction peak of the pure BiVO_4_ sample. Characteristic diffraction peaks in the spectrum observed at 28.822°, 28.947°, 28.586°, 18.669°, 18.988°, 30.548°, 54.243°, 46.711°, 47.305°, 34.494°, 35.221°, 39.782°, and 42.464° correspond to (−121), (121), (−130), (110), (011), (040), (−161), (240), (042), (200), (002), (211), and (051) diffraction planes of the BiVO_4_ crystal structure, respectively, which are marked with grey hearts. The data matches well with those in PDF#14-0688. The red line represents the diffraction peak of the pure In_2_O_3_ sample. Characteristic diffraction peaks in the spectrum positioned at 30.993°, 32.618°, 45.618°, 50.255°, 22.376°, and 57.205° correspond to the diffraction planes of the In_2_O_3_ crystal structure at (104), (110), (024), (116), (012), and (214), respectively. The data are well matched with those in PDF#22-0336. The blue line represents the diffraction peak of the In_2_O_3_/BiVO_4_ composite sample. It can be seen from the spectrum that besides the diffraction peak with the highest intensity, there are no impurity peaks, indicating that the sample exhibits high purity and high crystallinity. The characteristic diffraction peaks in the spectrum of the composite matched those of the pure sample, and the diffraction peak intensity of In_2_O_3_ in the composite sample was more pronounced compared with that of the pure sample. All the extra peaks were attributed to In_2_O_3_, indicating that the diffraction peaks of the In_2_O_3_/BiVO_4_ nanocomposites contained In_2_O_3_ and BiVO_4_ moieties, proving the successful preparation of the sample.

A detailed EDS profile of In_2_O_3_/BiVO_4_ is presented in Fig. 3(b), revealing the elemental distribution of the In_2_O_3_/BiVO_4_ nanocomposite. Notably, oxygen (O), vanadium (V), indium (In), and bismuth (Bi) are distinctly discernible; their respective weight percentages in the composite sample are 18.1%, 9.10%, 28.30%, and 44.50%.

### 3.2 Microstructure analysis of samples

**Fig. 4:**
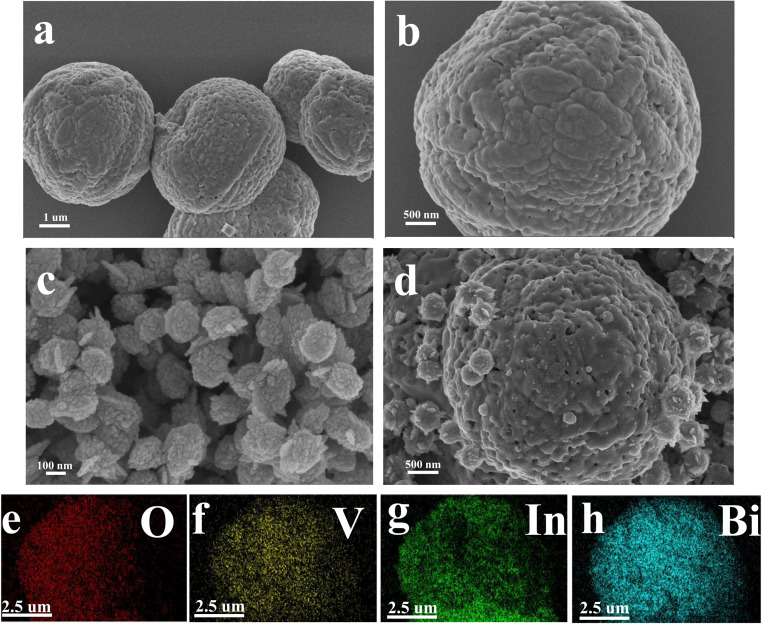
SEM images of the prepared samples: (a and b) BiVO_4_; (c) In_2_O_3_and (d) In_2_O_3_/BiVO_4_. (e–h) The element mapping of O, V, In, and Bi.

The microstructure of semiconductors is one of the important factors affecting the properties of materials. The particle size, morphology, porosity, specific surface area and microstructure of semiconductors are the main factors characterizing the sensitivity of sensors. Increasing the specific surface area of semiconductors will provide more active sites and higher activation energy for gas adsorption–desorption, thereby increasing the reaction rate. The compound can inhibit grain growth and reduce grain size, thereby increasing the specific surface area.^17^ The microstructures of BiVO_4_, In_2_O_3_ and In_2_O_3_/BiVO_4_ composites synthesized by employing a secondary hydrothermal method are shown in Fig. 4(a–d). In Fig. 4(a and b), the BiVO_4_ nanospheres synthesized *via* a hydrothermal method show a relatively uniform diameter. The nanospheres are spherical with a diameter of about 2 microns with many holes on its surface. It can be seen from Fig. 4(c) that the particle diameter of In_2_O_3_ is about 100 nanometers, which is very small compared to the diameter of BiVO_4_. Its surface is like a sea urchin, with the protrusions extremely dense. It can be seen from Fig. 4(d) that the In_2_O_3_/BiVO_4_ composite sample is composed of large and small balls with rough surfaces, where the large balls are BiVO_4_ nanospheres and the small balls are In_2_O_3_ nanospheres. The In_2_O_3_ small balls are evenly attached to the BiVO_4_ large balls, making the surface rougher and the holes more numerous. The increase in holes is attributed to numerous grain boundaries and defects caused by the two annealings, which improved the utilization rate of the sensing body and greatly improved the gas sensing performance. Fig. 4(e–h) are mapping images of the four elements O, V, In, and Bi of the In_2_O_3_/BiVO_4_ composite sample.^20^ Various elements are evenly distributed in the In_2_O_3_/BiVO_4_ composite sample. It can be seen from the SEM image that In_2_O_3_ and BiVO_4_ modify each other in the In_2_O_3_/BiVO_4_ composite material and finally form a self-consistent composite model.^21^

**Fig. 5:**
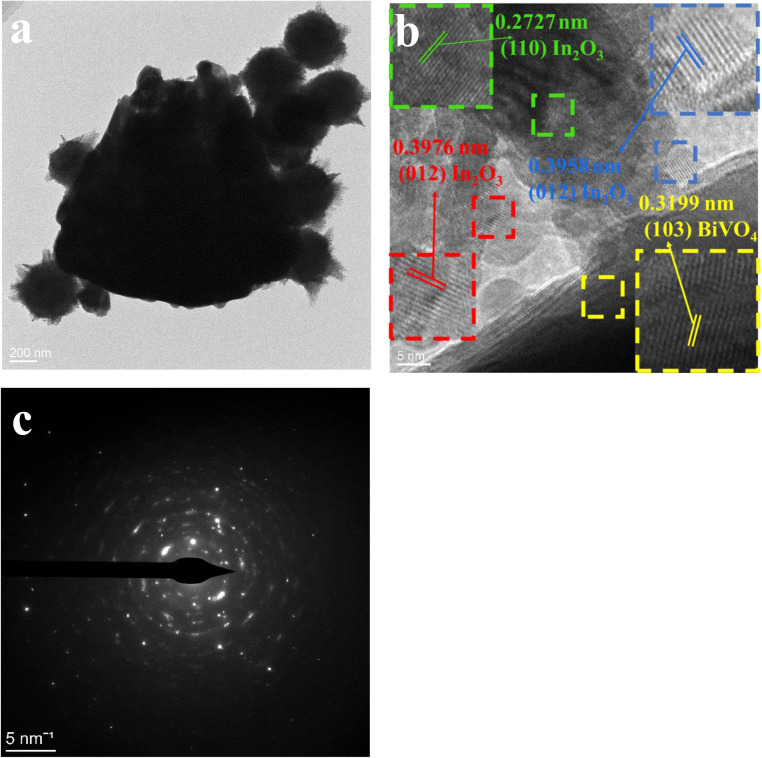
Image of In_2_O_3_/BiVO_4_: (a) TEM; (b) HRTEM; (c) SAED.

In Fig. 5(a), an evenly distributed TEM image exhibits In_2_O_3_ spheres on BiVO_4_'s surface. Both TEM and SEM analyses reveal the cohesive nano-spherical architecture of the composite. This unique pore design facilitates efficient gas molecule adsorption onto and diffusion through the In_2_O_3_/BiVO_4_ interface, enhancing material sensitivity. The high-resolution image in Fig. 5(b) allows for precise measurement of lattice spacings; the yellow BiVO_4_(103) plane measures 0.3199 nm, green In_2_O_3_(110) plane measures 0.2727 nm, while the blue and red In_2_O_3_(012) planes stand at 0.3958 and 0.3976 nm, respectively. The presence of lattice fringes in both materials in HRTEM analysis confirms the successful formation of the composite. Lastly, Fig. 5(c) presents a SAED pattern depicting a polycrystalline nature with excellent crystallinity, as evidenced by distinct concentric rings.

### 3.3 XPS characterization analysis of samples

The elemental composition and electronic states of the In_2_O_3_/BiVO_4_ composites were characterized using XPS. In Fig. 8(a), the V 2p peak of the XPS spectrum confirms the presence of V^5+^, where the V 2p_3/2_ and V 2p_1/2_ peaks are at 516.34 eV and 523.62 eV, respectively. In Fig. 8(b), the two peaks at 158.75 eV and 164.04 eV are attributed to Bi 4f_7/2_ and Bi 4f_5/2_, indicating the presence of Bi^3+^. In Fig. 8(d), the spectral In 3d peaks confirm the presence of In^3+^, in which the In 3d_5/2_ and In 3d_3/2_ peaks are at 444.44 eV and 451.95 eV, respectively.

Fig. 8(c) shows the high-resolution spectrum of O 1s. O 1s can be decomposed into three parts. The first peak at 529.62 eV corresponds to lattice oxygen (O_L_), the second peak located at 530.10 eV correspond to oxygen vacancies (the third peak position of O_V_), and 531.47 eV corresponds to adsorbed oxygen (O_C_). During high temperature calcination, atomic rearrangement leads to the formation of crystals. However, some weakly bonded oxygen atoms may leave their original position and become oxygen vacancies. More oxygen vacancies can provide more active sites for the adsorption of target molecules, promoting the reaction of gas molecules and oxygen, thereby improving the gas sensitivity properties of In_2_O_3_/BiVO_4_ composites.^22^

### 3.4 Spectral test and analysis of samples

Primarily, UV-visible absorption spectroscopy is deployed to analyze shifts in the absorption band edge and band gap, thus assessing the composite sample's influence on semiconductor light absorption. As illustrated in Fig. 7(a), distinctive curves denote the absorption spectra of In_2_O_3_ (blue), BiVO_4_ (red), and their blend In_2_O_3_/BiVO_4_ (black) across the wavelength range of 200–800 nm.

To conduct a more precise quantitative analysis, linear regression was applied to the slope of the absorption edge in Fig. 7(a), correlating photon energy and absorption coefficient. An extrapolation of the line to the *x*-axis enabled the determination of the sample's optical band gap. Similarly, in Fig. 7(b), blue, red, and black lines represent Ahv–Eg relationships derived from In_2_O_3_, BiVO_4_, and their composite at 200–800 nm, respectively. The optical band gaps were calculated as 3.08 eV for In_2_O_3_, 2.38 eV for BiVO_4_, and 3.18 eV for the In_2_O_3_/BiVO_4_ composite. This increased band gap, compared to that of the pure samples, is considered to be due to the fact that the formation of electron–hole pairs is affected by recombination, which alters the electronic structure and band gap of the material.

### 3.5 Study on the specific surface area and pore size distribution of samples

**Fig. 6:**
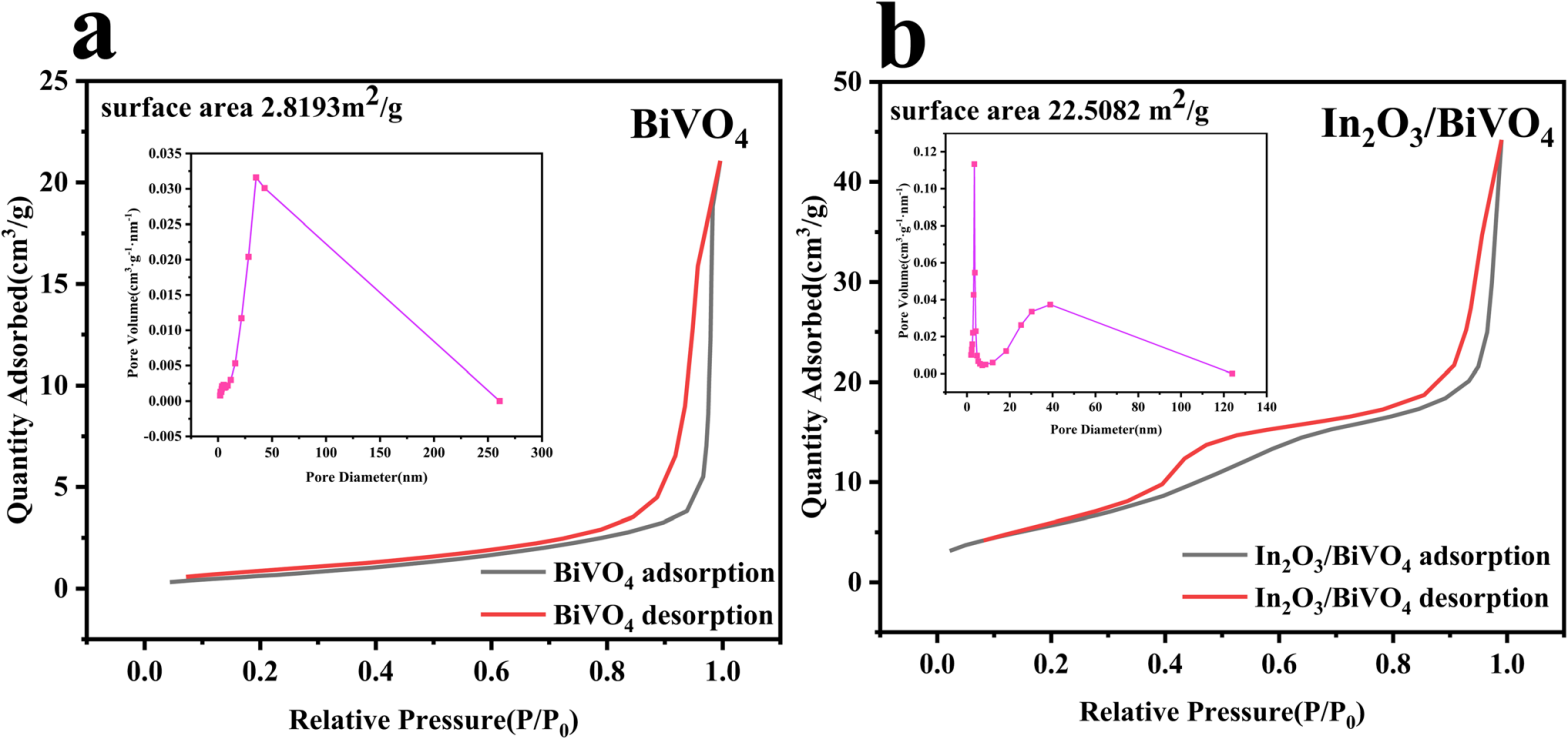
The adsorption and desorption isotherm and aperture curve of BiVO_4_ and In_2_O_3_/BiVO_4_: (a) BiVO_4_; (b) In_2_O_3_/BiVO_4_.

Utilizing nitrogen adsorption–desorption isotherm analysis, we examined the specific surface area and pore size distribution of both BiVO_4_ and the In_2_O_3_/BiVO_4_ composite, with results presented in Fig. 6.

**Fig. 7:**
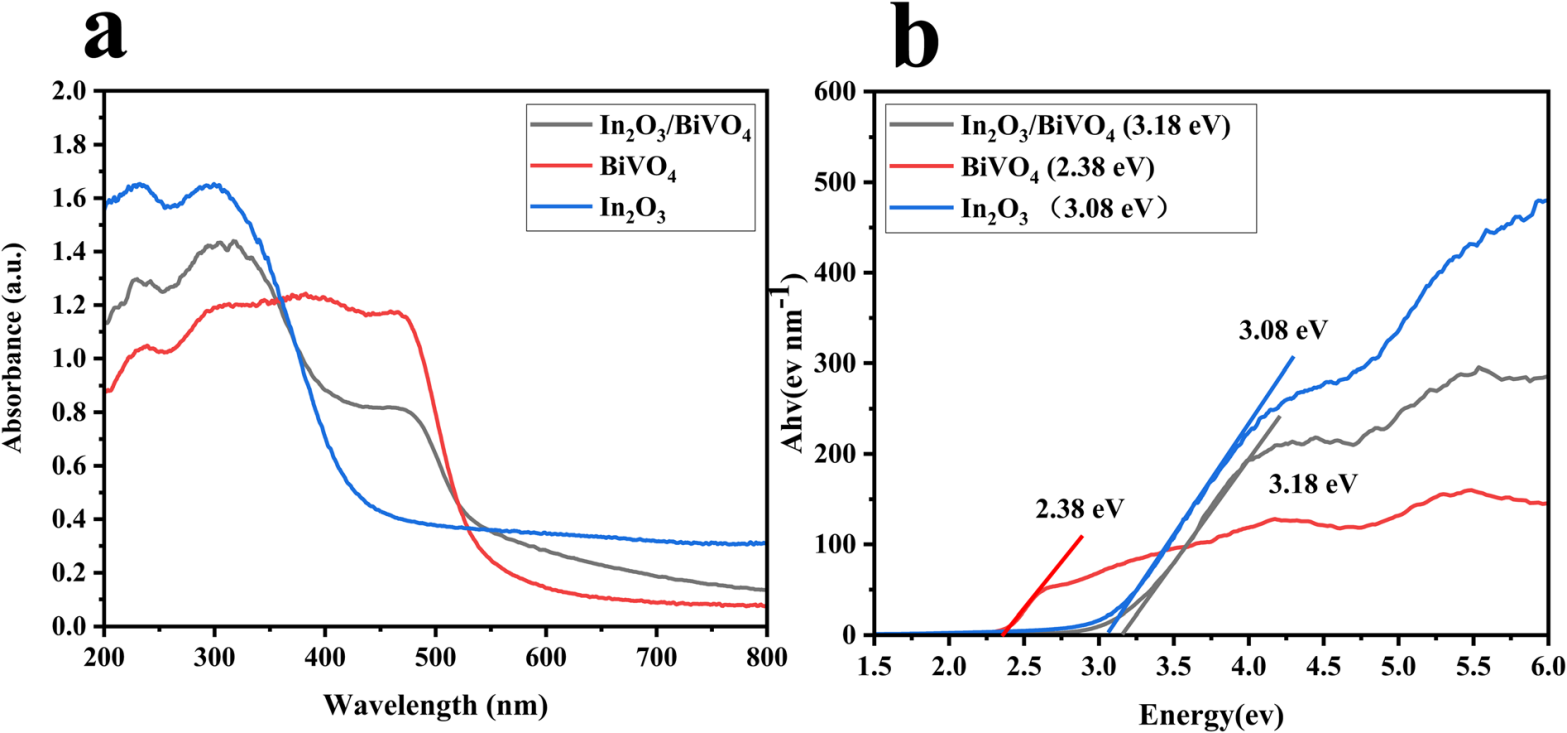
(a) The UV absorption spectra of In_2_O_3_, BiVO_4_ and In_2_O_3_/BiVO_4_. (b) The optical band-gap fitting curve of In_2_O_3_, BiVO_4_ and In_2_O_3_/BiVO_4_.

In Fig. 7(a) and (b), we present the N_2_ adsorption/desorption isotherms and corresponding Barrett–Joyner–Halenda (BJH) pore size distributions for both the pure mesoporous sample and nanocomposite. Notably, the nanocomposite exhibits clear hysteresis loops between 0.4 and 0.8 of *P*/*P*_0_, indicative of capillary condensation within its mesopores. Analyzing desorption data reveals a predominant pore size of 3.45 nm. The study of adsorption–desorption profiles uncovers a specific surface area of 2.8193 m^2^ g^−1^ for BiVO_4_ and a significantly enhanced value of 22.5082 m^2^ g^−1^ for the composite, suggesting efficient material blending and improved BiVO_4_ performance.

**Fig. 8:**
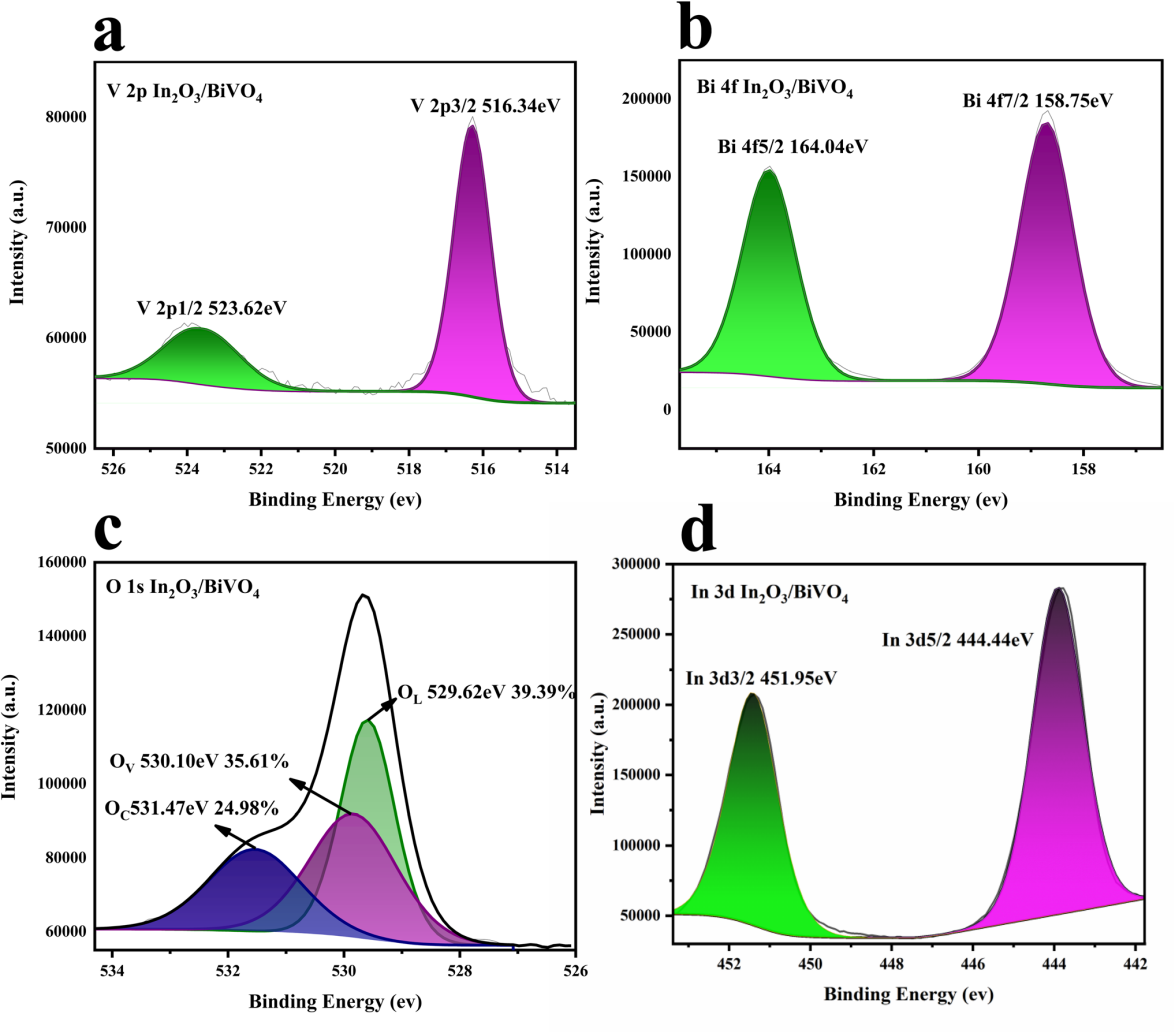
XPS spectra of In_2_O_3_/BiVO_4_: (a) V 2p peaks and (b) Bi 3f peaks; (c) O 1 s peaks and (d) In 3d peaks.

Enhancing the semiconductor surface area enhances reaction kinetics by offering more active sites and boosting adsorption–desorption energy. Grain boundary scattering in composites restrains grain growth, resulting in smaller grain sizes and increased surface area. Experimental data illustrate a substantial rise in the specific area and pore volume of In_2_O_3_/BiVO_4_. As observed in Fig. 6(a), pure BiVO_4_ exhibits 2.8193 m^2^ g^−1^, whereas In_2_O_3_/BiVO_4_ boasts 22.5082 m^2^ g^−1^ [Fig. 10(b)]. The composite's superior surface area is attributed to In_2_O_3_ spheres accumulating on BiVO_4_'s surface, creating roughness and porosity. BJH analysis in the inset of Fig. 6 reveals BiVO_4_'s pore size centered at 35.9 nm, compared to 3.337 nm for In_2_O_3_/BiVO_4_, indicating a denser pore structure. Combinatorial approaches, such as the synthesis of In_2_O_3_ and BiVO_4_ microspheres, are efficacious in generating diverse porous architectures, thereby significantly augmenting the specific surface area of the composite.

### 3.6 Study on gas sensitive properties of samples

To assess the gas sensing capabilities of the synthesized In_2_O_3_, BiVO_4_, and their composite In_2_O_3_/BiVO_4_ samples, we systematically performed exhaustive gas sensitivity tests, with the corresponding findings illustrated in Fig. 10 and 11.

**Fig. 9:**
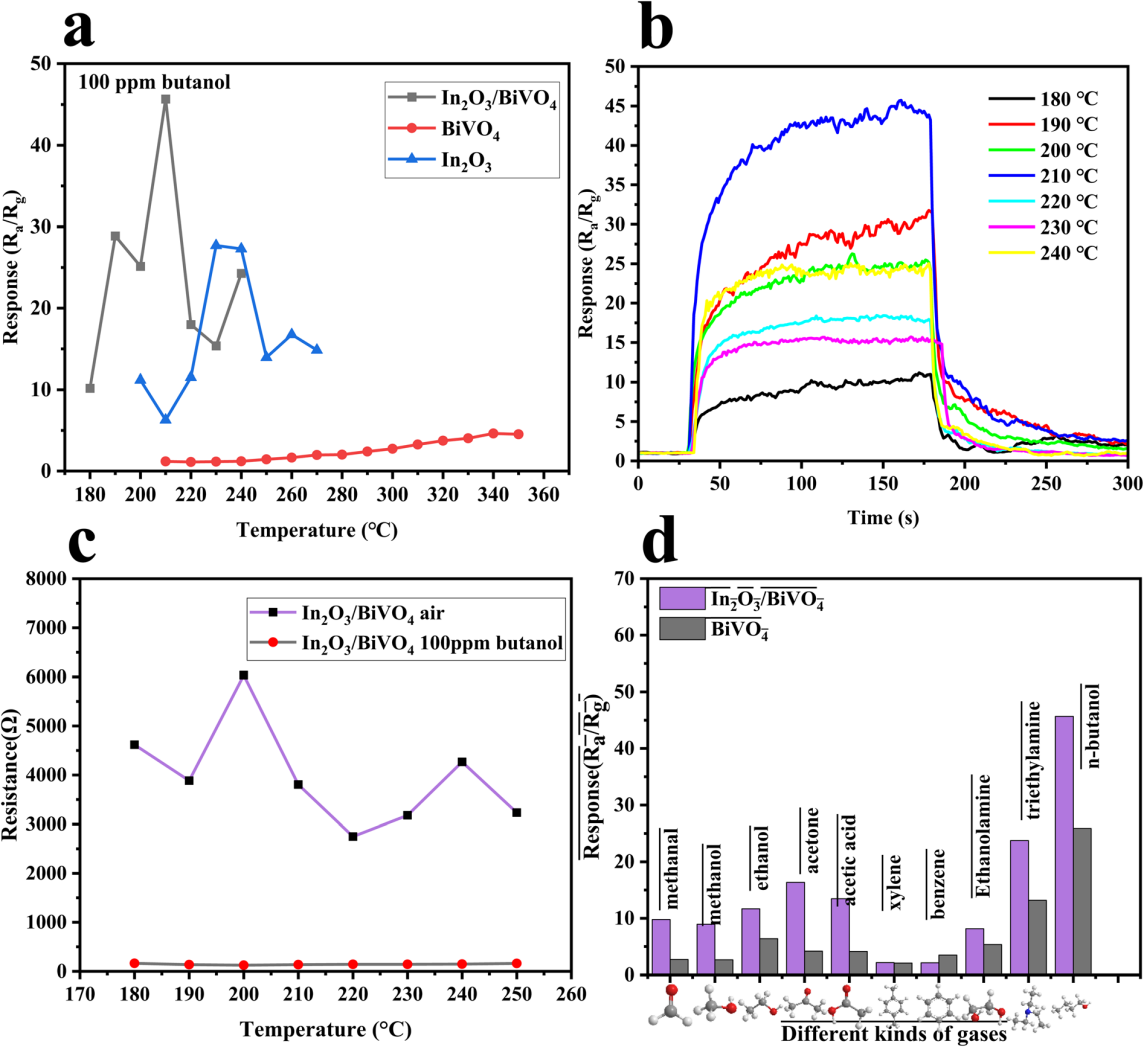
(a) Response of In_2_O_3_, BiVO_4_ and In_2_O_3_/BiVO_4_ to different temperatures at 100 ppm butanol. (b) Response of In_2_O_3_/BiVO_4_ at different temperatures to 100 ppm butanol. (c) Resistance of In_2_O_3_/BiVO_4_ in the air and butanol at different temperatures. (d) Selectivity test of BiVO_4_ and In_2_O_3_/BiVO_4_.

Operating temperature plays a key role in determining whether a sensor can be used in a certain environment. As shown in Fig. 9(a and b), the working temperature is 210 °C for In_2_O_3_/BiVO_4_ composites, 340 °C for pure BiVO_4_ materials and 240 °C for pure In_2_O_3_ materials, indicating that the working temperature of In_2_O_3_/BiVO_4_ composites is significantly lower than that of In_2_O_3_ and BiVO_4_. The resistance displayed in Fig. 9(c) increases steadily with temperature, reaching a peak before declining. This is due to the operational mechanism at lower temperatures: the formation of an n–n heterojunction in In_2_O_3_/BiVO_4_, facilitating electron transfer from the bandgap to the conductive band, thus lowering the sensor's required temperature.^23^

To prove the discriminatory power of the sample towards various gases, we identified the optimal target gas for the In_2_O_3_/BiVO_4_ sensor. By conducting tests at optimal conditions, we compared the sensor's responses (Fig. 9(d)) to 100 ppm of n-butanol, triethylamine, ethanolamine, benzene, xylene, acetic acid, acetone, ethanol, methanol, and formaldehyde. Notably, In_2_O_3_/BiVO_4_ exhibited superior selectivity for butanol, with a response value of 45.68, which is 1.5–2 times higher than that of other gases (13.18, 5.34, *etc.*). This result highlights the sensor's capability to selectively detect butanol.

**Fig. 10:**
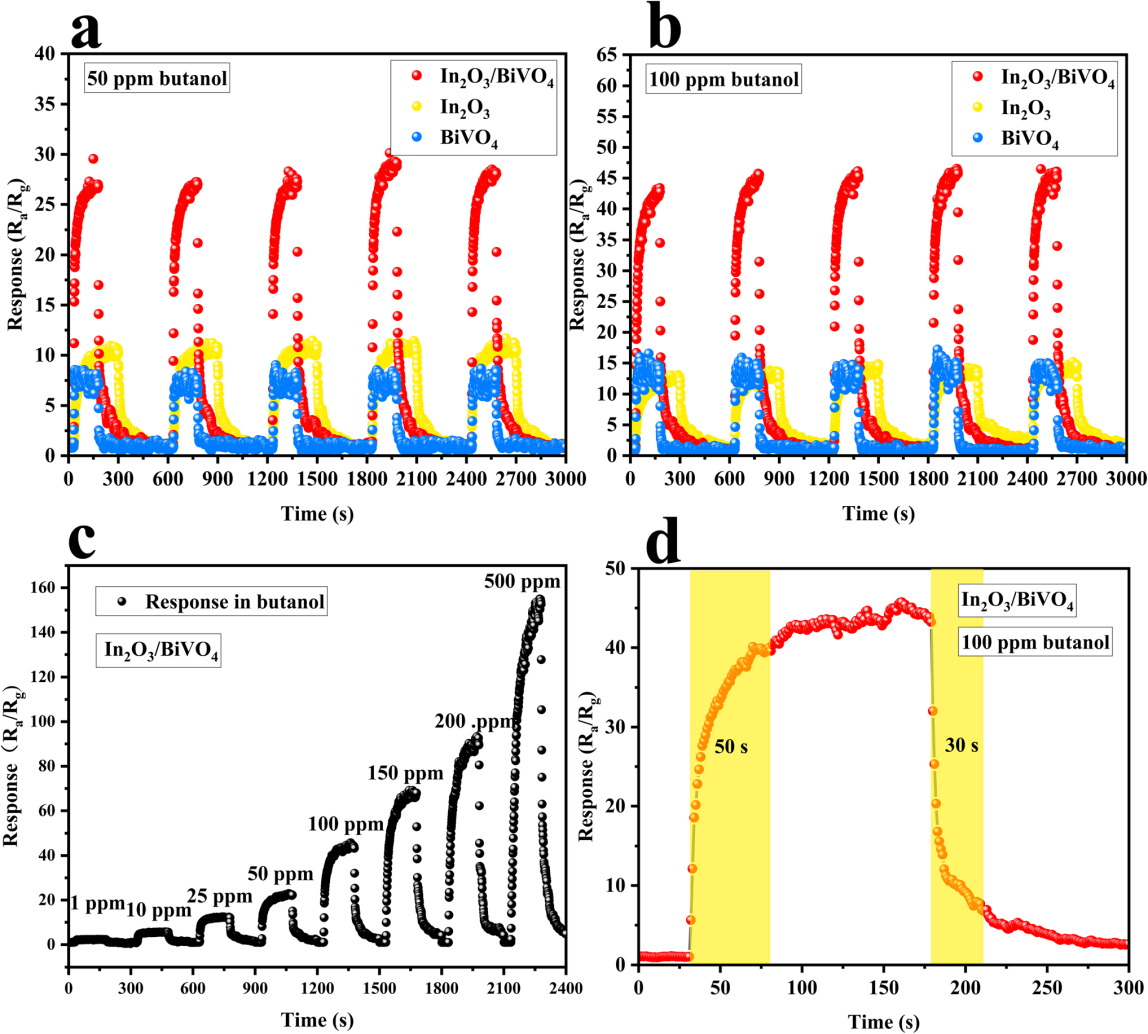
(a and b) Repeatable test responses of In_2_O_3_, BiVO_4_ and In_2_O_3_/BiVO_4_ to butanol at 50 ppm and 100 ppm. (c) Response of In_2_O_3_/BiVO_4_ to butanol at different concentrations. (d) Adsorption–desorption time of In_2_O_3_/BiVO_4_ at 100 ppm butanol.

As shown in Fig. 10(a) and (b), the repeated measurement plots of In_2_O_3_ and BiVO_4_ and their composite In_2_O_3_/BiVO_4_ were exposed to 50 ppm and 100 ppm of butanol at their respective optimal temperatures, and the composite consistently showed a significantly stable and improved response compared to the pure In_2_O_3_ and BiVO_4_ samples.

In order to evaluate the response to butanol gas at different concentrations, we conducted a dynamic test of the gas-sensitive response of the In_2_O_3_/BiVO_4_ composite sensor at an optimal temperature. As shown in Fig. 10(c), the test found that the response value of the sample increased with an increase in butanol concentration, and it was still unsaturated at 500 ppm. Furthermore, the dynamic response curve increased in a step-like manner. The response of the In_2_O_3_/BiVO_4_ composite sample to butanol was 2.17, 6.15, 12.36, 22.19, 44.87, 68.31, 92.96, and 154.91, corresponding to 1, 10, 25, 50, 100, 150, 200, and 500 ppm, respectively. Fig. 10(d) depicts the dynamic response–recovery curve of In_2_O_3_/BiVO_4_ towards 100 ppm butanol gas at 210 °C. It can be seen from the figure that the response time is 50 s, and the recovery time is 30 s.

**Fig. 11:**
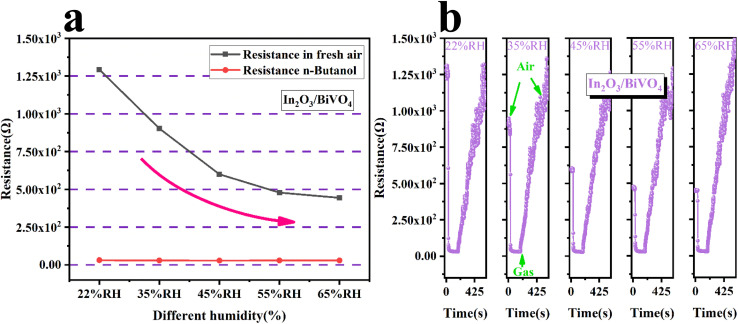
(a) Resistance of In_2_O_3_/BiVO_4_ in butanol and air at different humidities. (b) Resistance of In_2_O_3_/BiVO_4_ for 100 ppm butanol at different humidities.

Fig. 11(a) is a comparison of the resistance of In_2_O_3_/BiVO_4_ in butanol gas and the air at different humidities. It can be seen that the resistance of In_2_O_3_/BiVO_4_ in the air decreases with increasing humidity. The resistance of In_2_O_3_/BiVO_4_ in *n*-butanol increases with increasing humidity, but its upward trend is not significant, exhibiting only very subtle changes. The resistance of In_2_O_3_/BiVO_4_ in *n*-butanol is significantly lower than that in the air. This difference can be explained by the fact that the target gas competes with water vapor for the adsorption sites of the gas sensor. When humidity is low, active sites on the surface of In_2_O_3_/BiVO_4_ are mainly occupied by butanol molecules, and a small part is occupied by water molecules. As humidity gradually increases, water molecules occupy a large number of active sites for gas adsorption, gradually forming OH^−^ adsorbed on the surface of In_2_O_3_/BiVO_4_, blocking the adsorption of surface oxygen anions and the diffusion channels of butanol molecules, further hindering the contact between the gas and sensing materials and resulting in decreased sensitivity. Fig. 11(b) shows the resistance of In_2_O_3_/BiVO_4_ to 100 ppm butanol at different humidities.

**Fig. 12:**
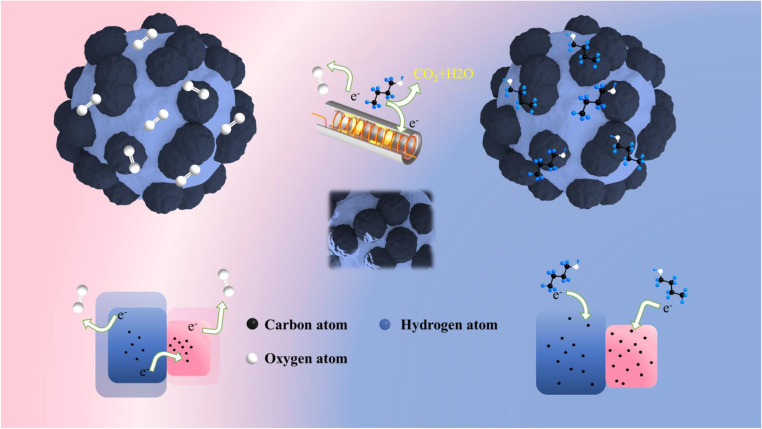
Gas-sensitive mechanism of In_2_O_3_/BiVO_4_ in the air and butanol.

In light of the experimental outcomes, our analysis delved into the butanol sensing behavior of In_2_O_3_/BiVO_4_ semiconductors. The prevalent sensing mechanism for n-type oxides revolves around the sensor's depletion zone modulation. As depicted in Fig. 12, employing 3D simulations, we showcased how this mechanism manifests as surface wrinkles in a ceramic tube sensor design. When butanol encounters adsorbed oxygen on the nanocomposite, it triggers redox reactions, producing electrons and holes. These charge carriers re-enter the conduction band, boosting electron density and, consequently, decreasing sensor resistance.

4 (C_4_H_9_OH)_gas_ → (C_4_H_9_OH)_ads_

5 (C_4_H_9_OH)_ads_ + 12O_ads_^−^ → 4 CO_2_ + 5H_2_O + 12e^−^

6 (C_4_H_9_OH)_ads_ + 12O_ads_^2−^ → 4 CO_2_ + 5H_2_O + 24e^−^

The main reasons for the enhanced gas sensing performance of In_2_O_3_/BiVO_4_ are summarized below:

(1) The compounding of In_2_O_3_ increases the surface roughness of the sample, the specific surface area of the sample, the number of surface atoms, surface activation energy, and the reaction rate.^24^

(2) The adsorbed oxygen ratio of the In_2_O_3_/BiVO_4_ composite increases, which provides more adsorption sites for the target gas and improves gas-sensing performance.^25^

(3) A secondary high-temperature hydrothermal process was employed to fabricate the n–n heterojunction, facilitating the development of an expanded depletion zone.^26^ This expansion enhanced the material's potential barrier, creating more room for electron transfer upon gas adsorption. Consequently, the increased resistance drop enabled the In_2_O_3_/BiVO_4_-based sensor to exhibit heightened sensitivity.

(4) The synergistic effect of In_2_O_3_ enhances its optical band gap in BiVO_4_ composites, improves electron transport efficiency owing to the effect of electron holes on recombination, and ultimately enhances gas sensing ability.

## 4. Conclusion

In summary, this experiment successfully synthesized In_2_O_3_/BiVO_4_ nanocomposites. Compared with pure In_2_O_3_ and BiVO_4_ samples, the selectivity and response value of the In_2_O_3_/BiVO_4_ nanocomposites to butanol gas were greatly improved. The optimal temperature of the In_2_O_3_/BiVO_4_ sensor was reduced to 210 °C, its response time was shortened to 50 s, and the sensitivity was stabilized at 45%. In addition, the composite demonstrated excellent resistance to humidity interference. The excellent gas-sensing performance of In_2_O_3_/BiVO_4_ is mainly attributed to the increase in the specific surface area, which improved response speed. At the same time, an n–n heterojunction was constructed, which effectively improved electron transfer efficiency, so that the target gas exhibited a sharp resistance change when attached to In_2_O_3_/BiVO_4_, thereby improving sensitivity. Therefore, the In_2_O_3_/BiVO_4_ composite is a promising candidate to improve the butanol sensing performance of BiVO_4_ and has great potential in preparing butanol sensors with high response and low detection limits.

## Data availability

All the data in this paper are measured using scientific instruments, and the model of instruments and equipment are provided in the experimental part. All the measured data are presented in the paper, and the data are real and reliable.

## Conflicts of interest

There are no conflicts to declare.
